# Health-related quality of life scores of metastatic pancreatic cancer patients responsive to first line chemotherapy compared to newly derived EORTC QLQ-C30 reference values

**DOI:** 10.1186/s12885-022-09661-7

**Published:** 2022-05-20

**Authors:** Suvina Amin, Seongjung Joo, Sandra Nolte, Hyun Kyoo Yoo, Nikunj Patel, Hilary F. Byrnes, Sara Costa-Cabral, Colin D. Johnson

**Affiliations:** 1 AstraZeneca, One Medimmune Way, Gaithersburg, MD 20878 USA; 2 Merck & Co, Inc, Kenilworth, NJ 07033 USA; 3 ICON Clinical Research GmbH, Konrad-Zuse-Platz 11, 81829 Munich, Germany; 4 Department of Psychosomatic Medicine, Charité – Universitätsmedizin Berlin, corporate member of Freie Universität Berlin and Humboldt-Universität zu Berlin, Medical Clinic, Berlin, Germany; 5 AstraZeneca, City house, 130 Hills road, Cambridge, CB2 1RE UK; 6 AstraZeneca, One Medimmune Way, Gaithersburg, MD 20878 USA; 7 ICON plc, 731 Arbor Way, Suite 100, Blue Bell, PA 19422 USA; 8 Mapi Research Trust, 27, Rue de la Villette, 3rd & 4th Floors, 69003 Lyon, France; 9 University of Southampton, University Road, Southampton, SO17 BJ UK

**Keywords:** Health-related quality of life, Patient-reported outcome, EORTC QLQ-C30, Reference values, Pancreatic carcinoma

## Abstract

**Background:**

Metastatic pancreatic cancer (mPC) and its treatments significantly impact health-related quality of life (HRQoL). POLO, a randomized, double-blind, placebo-controlled phase 3 trial evaluated the efficacy of olaparib as maintenance therapy in germline BRCA mutated mPC patients who had not progressed during ≥16 weeks of first-line platinum-based chemotherapy. HRQoL was assessed using the EORTC QLQ-C30. To enhance score interpretation, we derived reference values for treatment-naïve mPC patients from the literature.

**Methods:**

A targeted literature review identified EORTC QLQ-C30 baseline values in treatment-naïve mPC patients. Reference values were calculated by deriving means from studies meeting inclusion criteria, with scores from 0 to 100 (higher scores indicate better QoL/functioning but worse symptoms). For POLO patients, means were calculated using pooled baseline data across study arms.

**Results:**

Four studies met inclusion criteria. Depending on the specific scale, sample sizes ranged from *n* = 466 to *n* = 639. Compared to newly derived reference values, POLO patients reported markedly better HRQoL scores at baseline across most scales, with eight scales showing differences of ≥10 points. POLO patients’ HRQoL scores were often close to or better than general population norm data.

**Conclusions:**

This is the first study to systematically derive EORTC QLQ-C30 reference values for mPC. POLO patients had better HRQoL scores than those in the literature and similar to general population data. Comparatively high HRQoL of POLO patients are likely due to effects of prior first-line treatment and resolution of chemotherapy-related symptoms, response shift, or a combination. Newly derived reference values can enhance interpretation of mPC patients’ HRQoL.

**Trial registration:**

The POLO trial was registered on 9 July 2014 with ClinicalTrials.gov as NCT 02184195.

**Supplementary Information:**

The online version contains supplementary material available at 10.1186/s12885-022-09661-7.

## Background

Pancreatic cancer (PC) is a highly aggressive malignancy that frequently progresses to metastatic disease [1]. The majority of PC patients are not diagnosed until they have advanced disease and/or their disease has metastasized; only 15–20% are suitable for curative resection [2]. After diagnosis of metastases, survival is measured in months [3–5]. Therefore, treatment of metastatic pancreatic cancer (mPC) is aimed at symptom management, including aggressive treatment of pain [6] and maintaining health-related quality of life (HRQoL).

mPC is associated with symptoms such as pain, fatigue, reduced appetite and weight loss, and an overall decline in functional status [6, 7]. HRQoL of these patients is often poor [8–10], and may further be impaired by treatment [7, 11].

Palliative chemotherapy is the first-line treatment for mPC [12]. Treatment response or stabilization of disease may be achieved, but progression ultimately occurs [2, 13], with further impairment of HRQoL. Effective maintenance therapy for patients with responsive or stable disease would have the potential to maintain HRQoL.

The POLO trial was a randomized, double-blind, placebo-controlled phase 3 trial evaluating the efficacy of olaparib as maintenance therapy in germline BRCA mutated (gBRCAm) mPC patients who had not progressed during at least 16 weeks of first-line platinum-based chemotherapy. Patients reported their HRQoL via the QLQ-C30, of the European Organisation for Research and Treatment of Cancer (EORTC) [14]. HRQoL scores in POLO were high at baseline and remained high during maintenance treatment [14]. Patients in the POLO trial represent a group of patients with gBRCAm who responded to first-line platinum-based chemotherapy. This selection may have a substantial effect on HRQoL responses. The offer of maintenance therapy in the trial may further influence scores, and improve patients’ HRQoL.

The only potential source for HRQoL reference values is the EORTC QLQ-C30 reference values manual [15], yet these values were from a combined cohort of pancreatic, liver, and bile duct cancer patients, have not been updated in over 10 years and do not differentiate between patients with or without metastatic disease. As such, the validity of such comparison is in doubt. Disease-specific reference values derived from mPC patients after first-line treatment would offer the most accurate comparator but are lacking and would be difficult to obtain. Therefore the aim of the present study was to create up-to-date reference values for mPC patients and compare these to POLO data and general population norm data. This approach should improve interpretation of baseline data in the POLO trial.

## Methods

### Study design

The POLO trial was registered on 09/07/2014 with ClinicalTrials.gov as NCT 02184195. All study activities were performed in accordance with relevant guidelines and regulations, and approved by local ethics boards overseeing the study sites (see Supplemental Table 1 for the full list of IRBs and approval dates). Informed consent was obtained from all subjects and/or their legal guardian(s). The study consisted of three parts. First, reference values for the EORTC QLQ-C30 were derived from studies on mPC patients reported in the literature. Second, baseline scores from both the treatment and control arm of mPC patients in the POLO trial were calculated. Finally, the three sets of EORTC QLQ-C30 scores, i.e., newly derived mPC reference values, POLO baseline scores, and general population norm data [16] were compared.

### The EORTC QLQ-C30

The EORTC QLQ-C30 [17] assessed the QoL of cancer patients using 15 scales: five functional scales (physical, role, cognitive, emotional and social functioning); three symptom scales (fatigue, pain and nausea/vomiting); six single item scales (dyspnea, insomnia, appetite loss, constipation, diarrhea and financial difficulties); and a two-item global health status/QoL scale. The 28 items of the functional, symptom, and single item scales are rated on a four-point scale (i.e., 1 = not at all, 2 = a little, 3 = quite a bit, 4 = very much), while the two global health status/QoL items are rated on a seven-point scale (i.e., ranging from 1 = very poor to 7 = excellent). All scales are scored from 0 to 100, so that high scores for the global health status/QoL and functional scales reflect high/better functioning/QoL, but high symptom scale scores reflect increasing severity of symptoms/problems. Adequate reliability, validity, and sensitivity have been demonstrated [17–19]. A change in either direction of at least 10 points has been considered a clinically meaningful change [14, 20].

### General analysis considerations

The derivation of reference values was undertaken using MS Excel, while the analysis of POLO trial data was undertaken using SAS v9.4.

### Derivation of EORTC QLQ-C30 mPC reference values from the literature

The targeted literature search was performed using the MEDLINE and Embase databases on 3 March 2020. The targeted literature review was aimed at identifying published EORTC QLQ-C30 values as reported by mPC patients before first-line treatment. A literature review of articles in mPC published since 2005, using the search string shown in Table 1, was performed using MEDLINE and Embase databases (through OVID platform). Additional data sources included a review of the EORTC QLQ-C30 reference values manual [15] as well as trial data. The review of clinical trials was performed in ClinicalTrials.gov (Advanced search engine) with the following search strategy: Pancreatic Neoplasms (condition or disease), EORTC QLC-C30 (outcomes), Phases 2, 3, 4, and not applicable, and time limit was set to: 01/01/2005 to date.

**Table 1:** Applied search string in MEDLINE and Embase literature review

Search #	Strategy
1	*Disease terms:* exp. Pancreatic Neoplasms OR (Cancer of Pancreas or Cancer of the Pancreas or Neoplasms, Pancreatic or Pancreas Cancer or Pancreas Neoplasms or Pancreatic Cancer).ab,ti.
2	*Questionnaire terms:* ((European Organization for Research and Treatment of Cancer quality of life questionnaire) or EORTC QLQ-C30 or EORTC QLQ C30 or EORTC C30 or EORTC-C30).ab,ti.
3	#1 AND # 2
4	#3 AND Limits: Abstract, English language, past 15 years (“2005-current”)

The subsequent screening process followed pre-defined inclusion criteria developed by the study team in collaboration with three scientific leaders participating in the study (HLK, CJ, and Andrew Bottomley). Studies including mixed cancer stages, mixed cancer types, patients receiving prior treatment, and those that reported medians only rather than mean values of EORTC QLQ-C30 scores were excluded. Where reported, reference values were derived for all EORTC QLQ-C30 scales. The calculation of mean baseline scores was carried out by first weighting the study means by the respective study’s sample size.

### Analysis of POLO trial data

The POLO trial included 154 patients with mPC. Simple mean scores based on the pooled dataset were calculated for the comparison with the newly derived EORTC QLQ-C30 reference values.

### Comparison of mPC reference values with general population norm data

After deriving new EORTC QLQ-C30 reference values for mPC from the literature and calculating POLO trial baseline values, results were compared. In addition, we compared POLO trial scores to norm data that had been published previously based on *N* = 11,343 persons from the European general population [16]. We applied published criteria [20, 21] to determine the size and clinical meaningfulness of any difference in scores between POLO data, the newly derived reference values, and general population norm data.

## Results

### Literature review

**Fig. 1:**
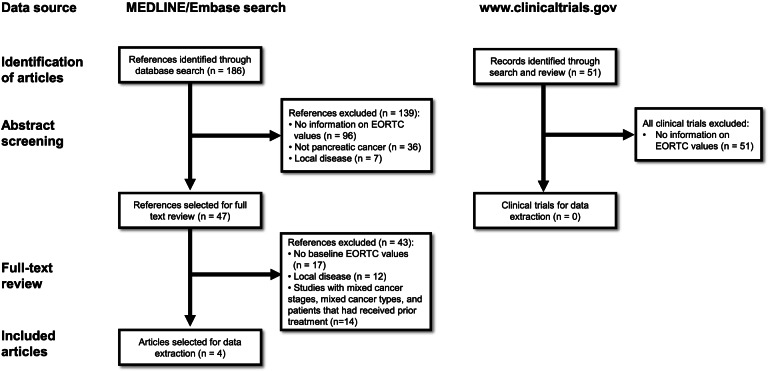
PRISMA Flow Diagram

Out of 186 articles identified, 47 references were selected for full text review (see Fig. 1). After applying all a priori defined exclusion criteria, four studies were included [10, 22–24].

A review of www.clinicaltrials.gov was performed, but no relevant data was found.

### EORTC QLQ-C30 reference values for mPC

As shown in Table 2, sample sizes for the new mPC reference values ranged between *n* = 466 (financial difficulties) and *n* = 639 (physical functioning, fatigue, pain) depending on respective EORTC QLQ-C30 scale.

**Table 2:** Comparison of newly derived EORTC QLQ-C30 reference values for metastatic pancreatic cancer, baseline QLQ-C30 POLO scores, and QLQ-C30 general population norm data

EORTC QLQ-C30 Scale	Newly derived mPC reference values		Baseline POLO scores (*n* = 147)	General population norm data^1^	Difference^2^ (POLO vs. mPC reference values)	Difference^2^ (mPC reference values vs norm data)	Difference^2^ (POLO vs. general population norm data)
	n	Mean	Mean	Mean	Mean difference	Mean difference	Mean difference
Global health status/QoL	629	54.3	71.9	66.1	17.6	−11.8	5.8
Functional scales							
Physical functioning	639	78.2	83.9	85.1	5.8	−6.9	−1.2
Role functioning	473	62.5	78.1	84.3	15.6	−21.8	−6.2
Emotional functioning	638	65.9	81.4	74.2	15.5	−8.3	7.2
Cognitive functioning	473	81.5	85.5	84.8	4.0	−3.3	0.7
Social functioning	469	70.2	76.8	86.2	6.6	−16.0	−9.4
Symptom scales							
Fatigue	639	46.0	29.5	29.5	−16.5	16.5	−0.0
Nausea and vomiting	476	16.2	8.3	5.9	−7.9	10.3	2.4
Pain	639	41.9	16.6	23.5	− 25.3	18.4	−7.0
Dyspnea	475	19.4	10.0	15.9	−9.4	3.5	−5.9
Insomnia	476	41.6	23.1	26.6	−18.5	15.0	−3.5
Appetite loss	476	44.6	14.7	10.0	−29.9	34.6	4.7
Constipation	473	32.7	13.4	12.5	−19.3	20.2	0.9
Diarrhea	469	13.5	15.5	9.5	2.0	4.0	6.0
Financial difficulties	466	15.7	19.3	10.6	3.6	5.1	8.7

^1^The “EORTC QLQ-C30 Norm” is based on *n* = 11,343 persons from the general population of 11 European countries [16]

^2^Clinically meaningful difference:10 points [14]

Data from patients with mPC yielded low scores (i.e., poor HRQoL) for global health status/QoL (54.3; on a scale 0–100) and role functioning (62.5), whilst high scores (i.e., good HRQoL) were observed for cognitive (81.5) and physical (78.2) functioning. For symptom scores, including financial difficulties, high scores (i.e., severe symptoms) were observed for fatigue (46.0), pain (41.9), insomnia (41.6), and appetite loss (44.6). Lowest scores (i.e., good HRQoL) were observed for diarrhea (13.5), financial difficulties (15.7), and nausea/vomiting (16.2).

### Comparison of EORTC QLQ-C30 reference values for mPC with general population norm data

Consistent with clinical experience, all scales for the newly derived EORTC QLQ-C30 reference values for mPC indicated worse HRQoL compared to general population norm data. Global health status/QoL and two functional scales (role and social functioning) showed differences of ≥10 points, indicating worse QoL/functioning reported by patients contributing data to the newly derived reference values. In addition, six symptom scales (fatigue, nausea and vomiting, pain, insomnia, appetite loss, and constipation) showed differences of ≥10 points, indicating more severe symptoms for the newly derived reference values compared to norm data. Of these differences, seven scales (role functioning, social functioning, fatigue, pain, insomnia, appetite loss, and constipation) showed large differences of ≥15 points.

### POLO trial EORTC QLQ-C30 scores

POLO trial EORTC QLQ-C30 mean scores are shown in Table 2. Within this group, the lowest functional scores (i.e., worse HRQoL) were for global health status/QoL (71.9) and social functioning (76.8), and the highest scores were observed for cognitive (85.5) and physical (83.9) functioning. For symptom scores, POLO patients reported high (worse) scores for fatigue (29.5), insomnia (23.1), and financial difficulties (19.3). The lowest symptom scores were observed for nausea/vomiting (8.3) and dyspnea (10.0).

### Comparison of POLO EORTC QLQ-C30 baseline scores with EORTC QLQ-C30 reference values for mPC

**Fig. 2:**
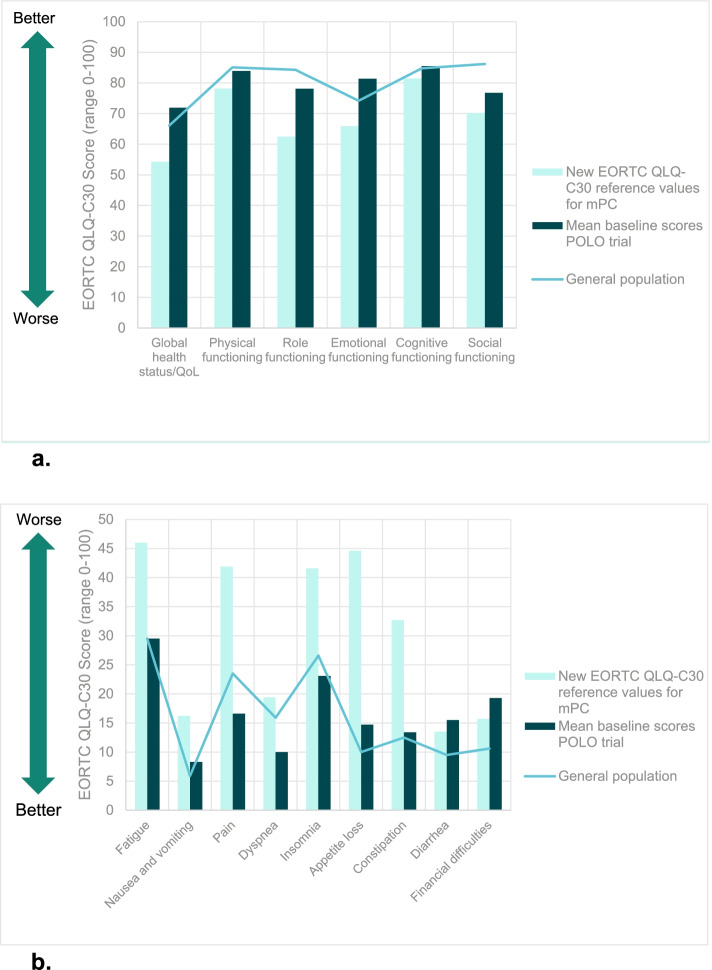
**a** EORTC QLQ-C30 scale scores, reference values, and general population scores versus POLO mean baseline scores, Global health status/QoL and functional scales. Clinically meaningful difference:10 points [14]. **b** EORTC QLQ-C30 scale scores, reference values, and general population scores versus POLO mean baseline scores, symptom scales Clinically meaningful difference:10 points [14]

When compared to the newly derived EORTC QLQ-C30 reference values for mPC, POLO patients reported markedly better scores in eight of the 15 scales when applying a threshold of ≥10 points (higher for global health status/QoL and functioning, and lower for symptoms, Table 2, Fig. 2a and b). Global health status/QoL was reported 17 points higher by POLO patients than in the mPC reference values. Large differences were also seen in role and emotional functioning (> 15 points higher in the POLO group), and in symptom severity (> 15 points lower for pain, fatigue, insomnia, appetite loss, and constipation).

### Comparison of POLO EORTC QLQ-C30 baseline scores and EORTC QLQ-C30 general population norm data

POLO patients reported overall similar HRQoL scores to the general population norm data, with none of the differences reaching the threshold of 10 points (Table 2, Fig. 2a and b). Role and social functioning, and diarrhea and financial difficulties were marginally worse in POLO patients, whereas global health status/QoL, emotional functioning, pain, and dyspnea showed differences of between 5.8 and 7.2 points in favor of POLO trial patients indicating marginally better HRQoL in these domains among POLO patients. The remaining scales, with differences < 5 points, indicated that prior to their maintenance therapy, POLO patients reported HRQOL similar to that reported by the general population.

## Discussion

This is the first study to systematically derive HRQoL reference values based on the EORTC QLQ-C30 to facilitate interpretation of patient-reported outcomes in mPC. The EORTC QLQ-C30 reference values manual [15] combines data from patients with or without metastatic disease as well as pancreatic, liver, and bile duct cancer, which limits its interpretability. The newly derived reference values, along with general population norm data [16], greatly enhance interpretability of HRQoL data obtained from mPC patients participating in clinical trials or other cross-sectional or longitudinal research. HRQoL data from the POLO trial suggested preservation of overall QoL during olaparib treatment, similar to placebo [14].

As expected, pooled baseline HRQoL scores for POLO patients demonstrated better HRQoL (i.e., higher functioning, lower symptom levels) compared with mPC patients in the literature. While newly derived reference values were based on treatment-naïve mPC patients, as opposed to patients who had shown response to previous treatment like POLO trial patients, observed differences between baseline POLO data and newly derived reference values are still substantially larger than expected. That is, the magnitude of differences in over half of the EORTC QLQ-C30 scales exceeded 15 points (see Table 2), which is well beyond what would be considered a clinically meaningful difference [20, 21].

The largest differences were for the appetite loss and pain scales, for which POLO baseline scores were over 25 points better than the newly derived reference values. This was further supported by the observation that baseline HRQoL scores of POLO patients were more in line with general population norm data [16] as opposed to mPC reference values. For example, differences from norm data scores never exceeded 10 points across all EORTC QLQ-C30 scales, with scales for global health status/QoL, emotional functioning, pain, and dyspnea even showing differences between 5 and 10 points in favor of POLO patients. Baseline HRQOL in POLO was marginally worse (differences of more than five but less than 10 points) than norm data only for role and social functioning, diarrhea, and financial difficulties scale scores. This suggests that POLO patients, who had stable disease after first-line platinum therapy and were expecting to receive maintenance therapy, reported HRQoL at similar levels to a normal population. Explanations for this finding could be the control of symptoms by chemotherapy, resolution of chemotherapy-related symptoms influencing patient perceptions, and/or increased hope related to anticipated benefit from planned maintenance therapy.

The newly derived reference values also provide a valuable update to reference values provided in the EORTC QLQ-C30 Reference Values manual [15], as the manual pooled values from pancreatic, liver, and bile duct cancer patients, as well as patients with or without metastatic disease, and has not been updated in more than 10 years. While the global health item and functional scale scores are similar to those reported in the EORTC QLQ-C30 Reference Values manual, several symptom scores show substantial discrepancies. Specifically, the newly derived reference values for mPC patients are more than 10 points higher (i.e., indicating more severe symptoms) than those in the manual for pain, appetite loss, and constipation.

Observed differences between baseline HRQoL scores from the POLO trial and reference values – and the close proximity of POLO data to general population norm data – further suggest that at least some patients in the POLO trial may have experienced changes in internal standards and values during or shortly after concluding platinum-based chemotherapy [25]. Such response shifts – that can be catalyzed by a cancer diagnosis or treatment – have an impact on the validity of measuring HRQoL, in particular, measuring change over time if patients undergo a response shift between time points [26]. Experiencing side effects of chemotherapy might lead patients to reprioritize different aspects of QoL, reevaluate the meaning of QoL constructs, and recalibrate response scales.

The possible contribution of psychological factors related to therapy may also contribute to response shift. Low pain levels after chemotherapy might point to recalibration response shift, i.e., any alleviation of pain is valued highly by mPC patients even if – objectively – their pain may not be as low as general population levels, even though our findings suggest that this was indeed the case. Such unexpected findings, i.e., good HRQoL of severely ill patients, have been described as “the disability paradox” [27], which may have been experienced by some POLO patients as well.

Our study has limitations. Although our reference values for mPC are derived from large patient numbers, these were treatment-naïve patients, as data were not available for the group with stable disease after first-line chemotherapy. It is important to note that there is a lack of data on HRQoL in the further course of maintenance therapy, as baseline data was examined in the current study. This may limit comparability of these values with POLO trial patients, as the latter group had experienced positive effects of chemotherapy. Our study design accepted this necessary compromise in order to provide the best available comparator group to improve understanding of POLO data. Reference values based on treatment-naïve mPC patients will have greater applicability to other studies of first-line or maintenance therapy. Future studies deriving reference values during maintenance therapy would be valuable.

Second, we applied rather strict inclusion criteria; however, we are convinced that a pure sample of mPC patients, i.e., no mixing of cancer stages or other cancer types, was appropriate in this context to enable as accurate a comparison as possible. Also, as our sample sizes were sufficiently large, applying strict inclusion criteria did not come at the expense of compromising the robustness of the derived reference values.

The newly derived EORTC QLQ-C30 reference values for mPC enhance interpretation of patients’ HRQoL both at true baseline, i.e., at the start of first-line treatment, but also at the start of maintenance treatment. Understanding the relative level of mPC patients’ HRQoL also greatly helps with the interpretation of HRQoL scores over time.

## Conclusions

mPC and its treatments significantly impact patients’ HRQoL. We systematically derived EORTC QLQ-C30 reference values for mPC based on treatment-naïve mPC patients as reported in the literature. Compared to newly derived reference values, the better baseline HRQoL scores in the POLO trial are likely due to positive effects of prior first-line treatment and resolution of chemotherapy-related symptoms, response shift, or a combination of these. These newly derived reference values, in combination with norm data, can enhance the interpretation of mPC patients’ HRQoL scores in first-line or maintenance treatment settings.

## Supplementary Information

**Additional file 1.**

## Data Availability

The datasets generated and/or analyzed during the current study are not publicly available to protect patient privacy, but are available from the corresponding author on reasonable request. Qualified researchers can request access to anonymized individual patient-level data from AstraZeneca group of companies sponsored clinical trials via the request portal. All requests will be evaluated as per the AZ disclosure commitment: https://astrazenecagrouptrials.pharmacm.com/ST/Submission/Disclosure.

## Abbreviations

mPC: Metastatic pancreatic cancer
HRQoL: Health-related quality of life
EORTC: European Organisation for Research and Treatment of Cancer
QoL: Quality of Life
