# Supplementary figures and images for: Health-related quality of life scores of metastatic pancreatic cancer patients responsive to first line chemotherapy compared to newly derived EORTC QLQ-C30 reference values
Source: BMC Cancer. 2022 May 20;22:563. doi: 10.1186/s12885-022-09661-7 (PMC9123808; doi:10.1186/s12885-022-09661-7)

**Supplemental Table 1**


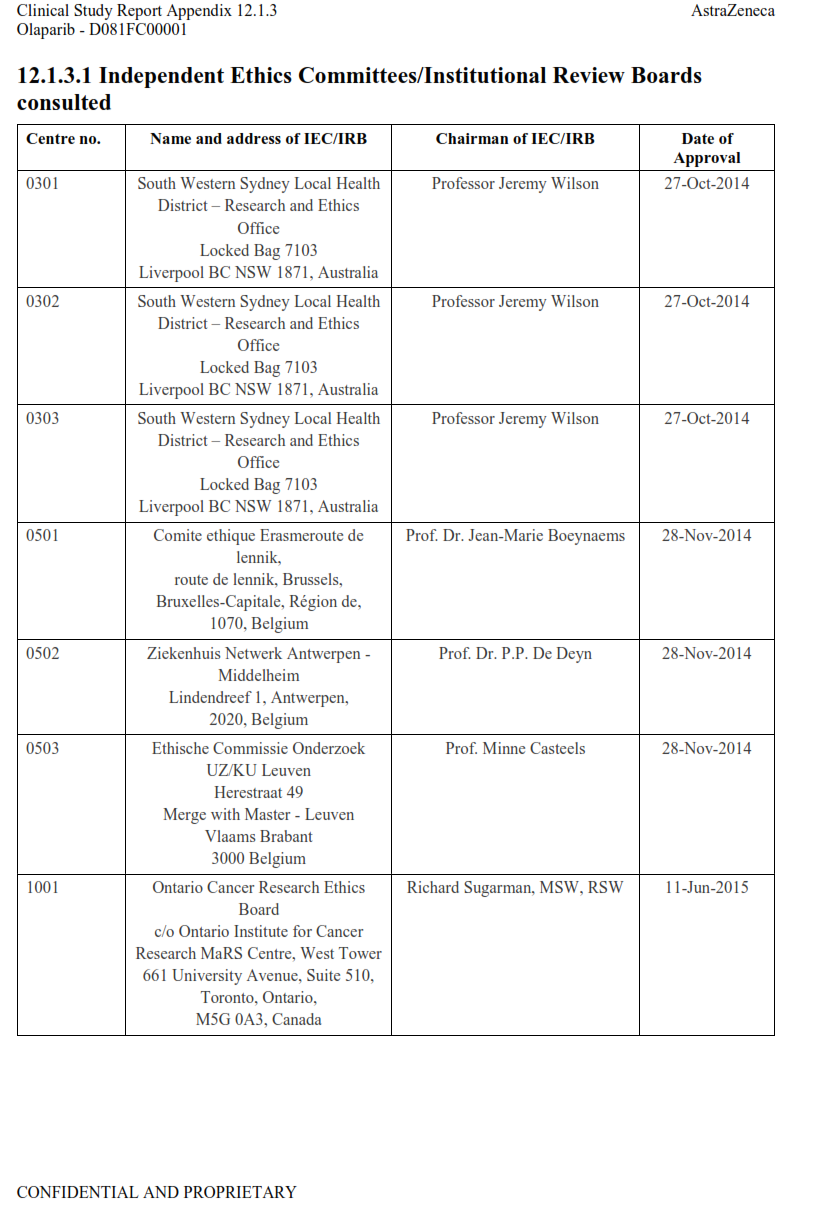

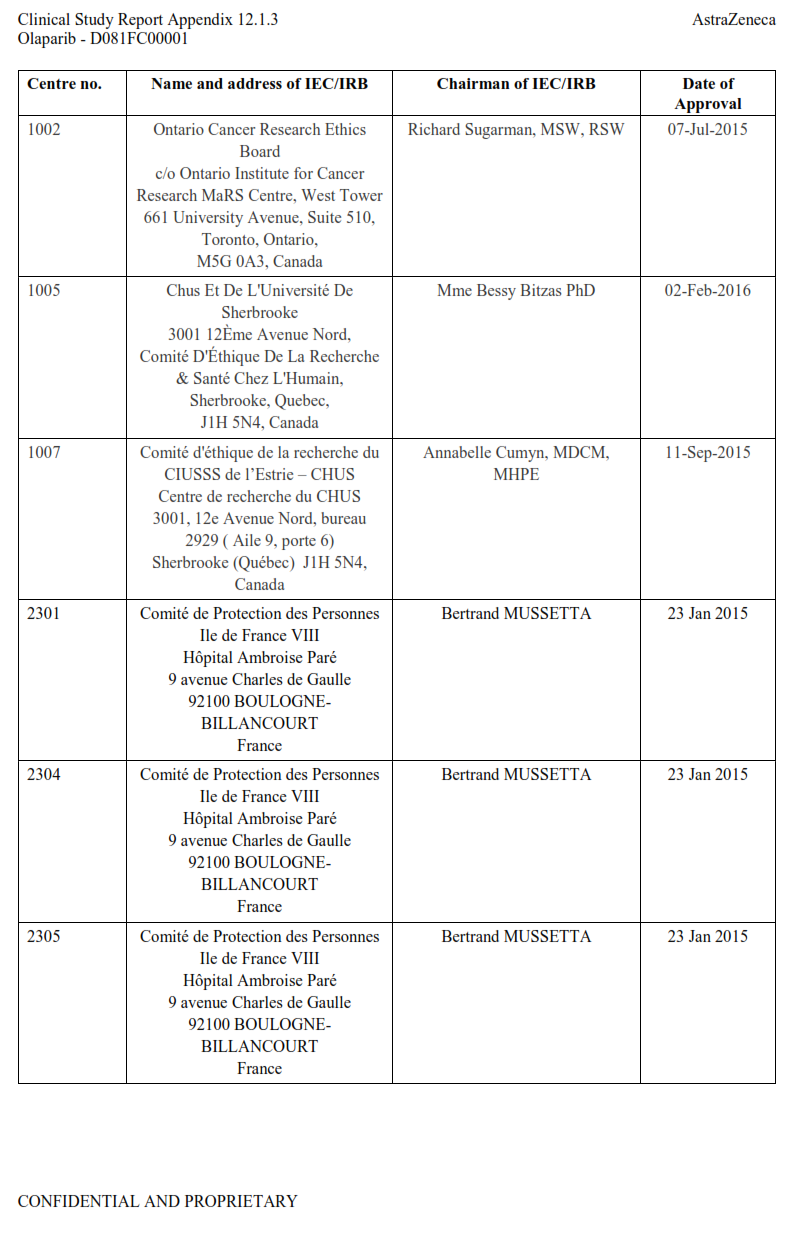

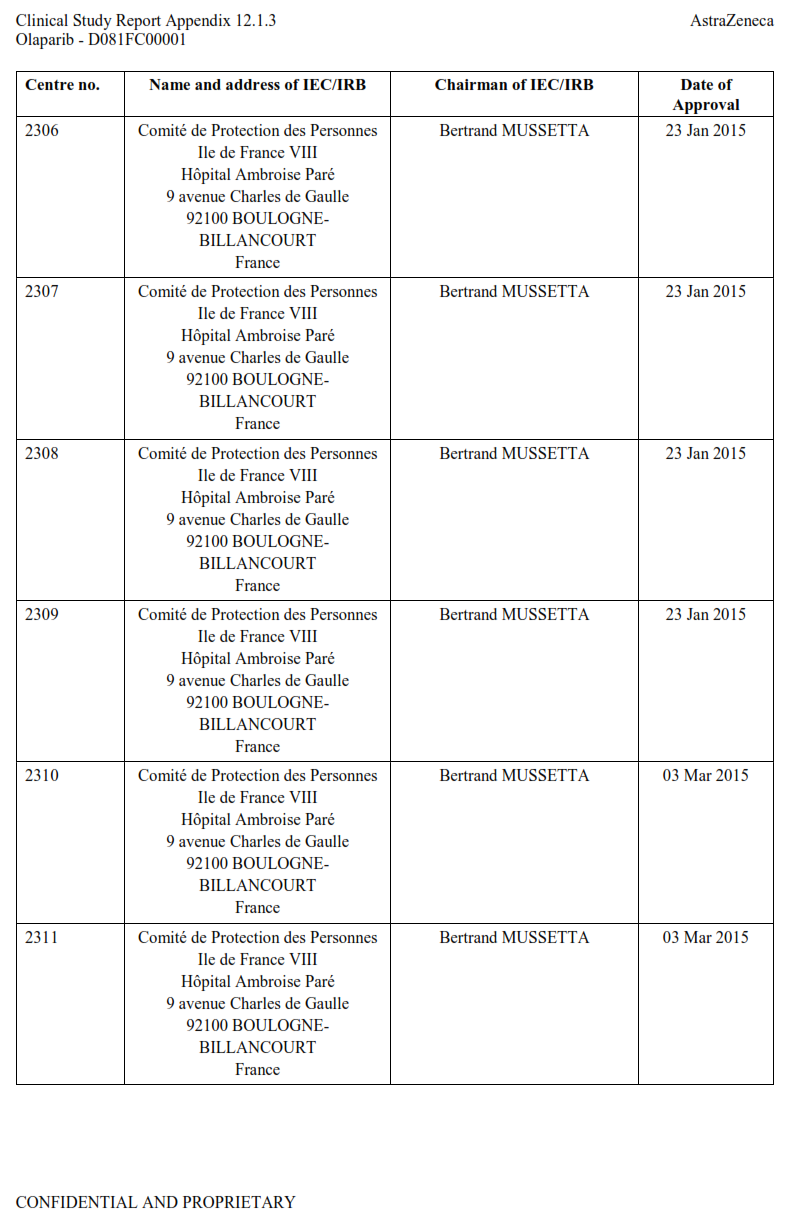

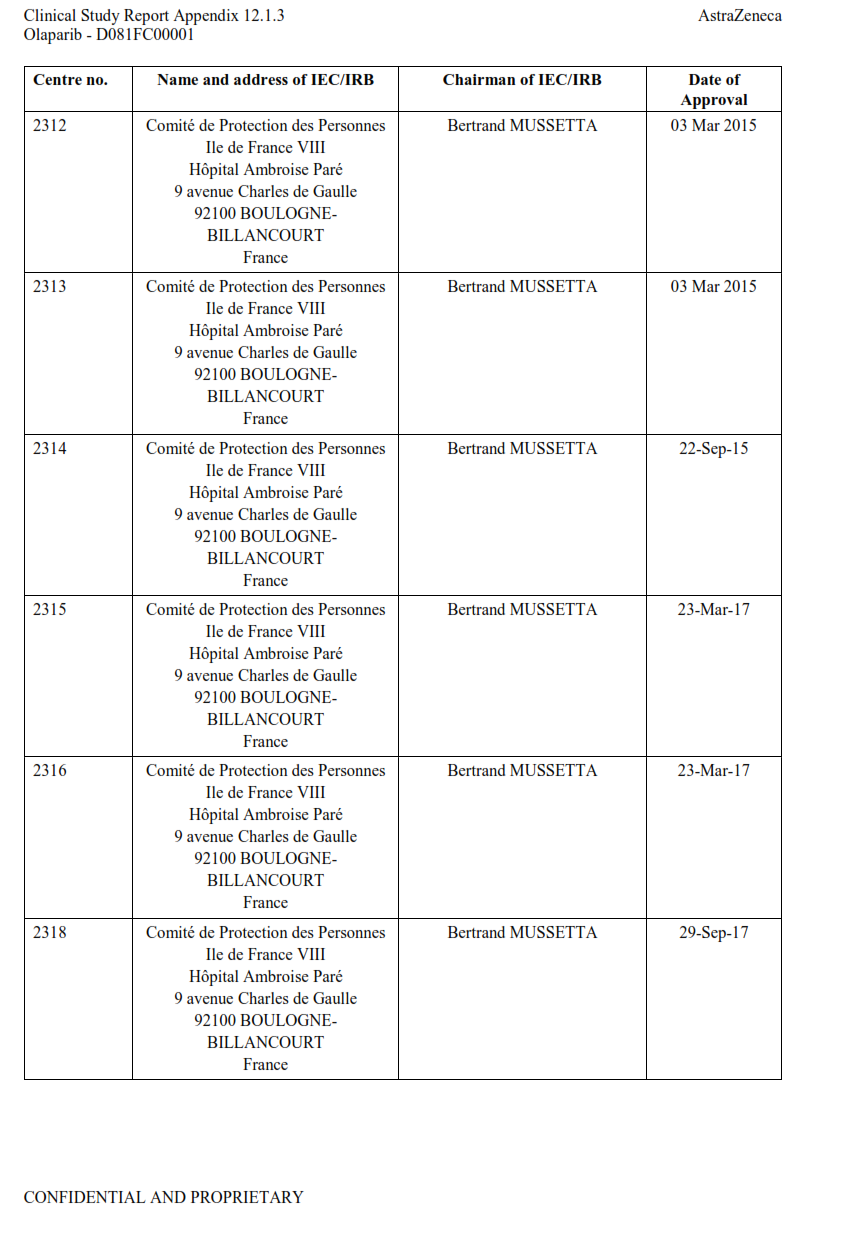

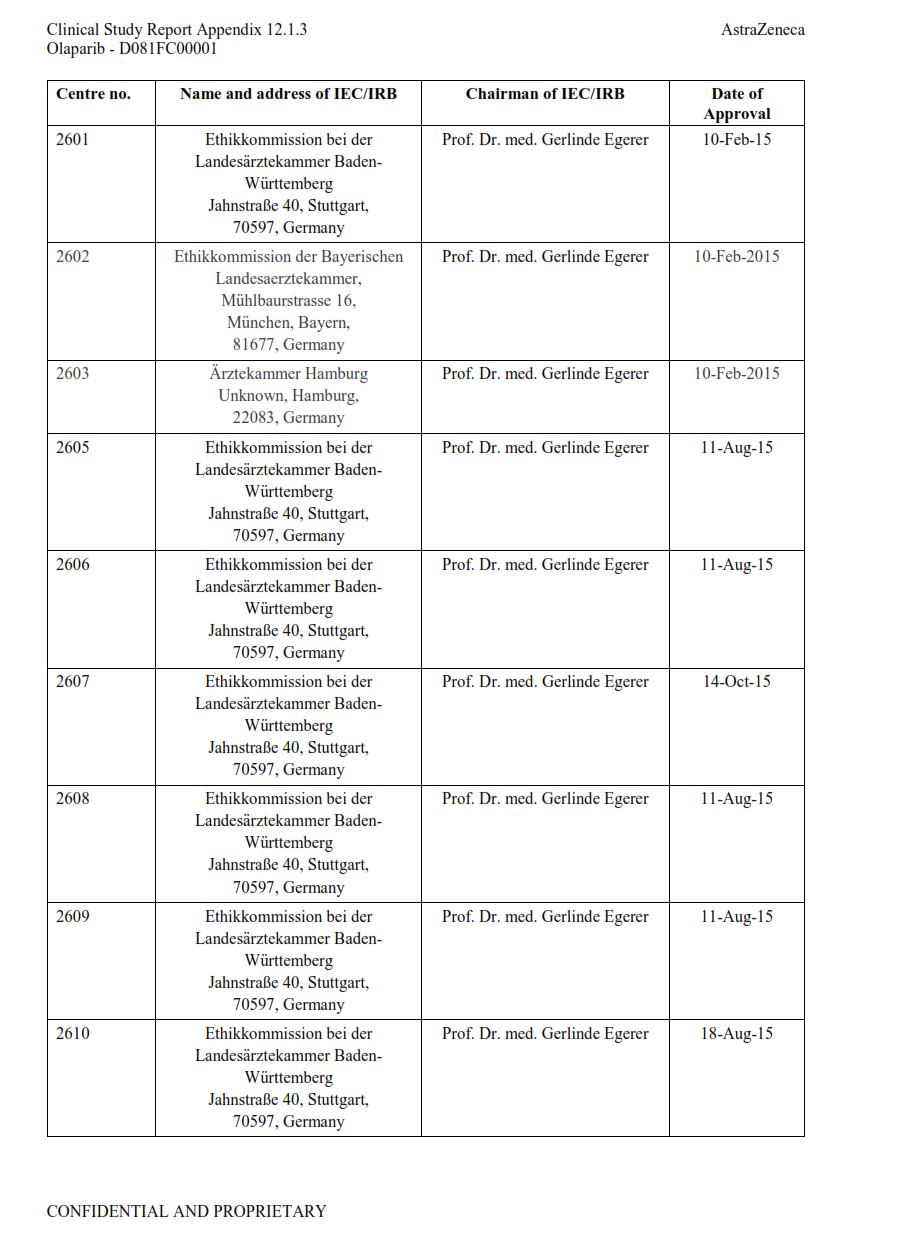

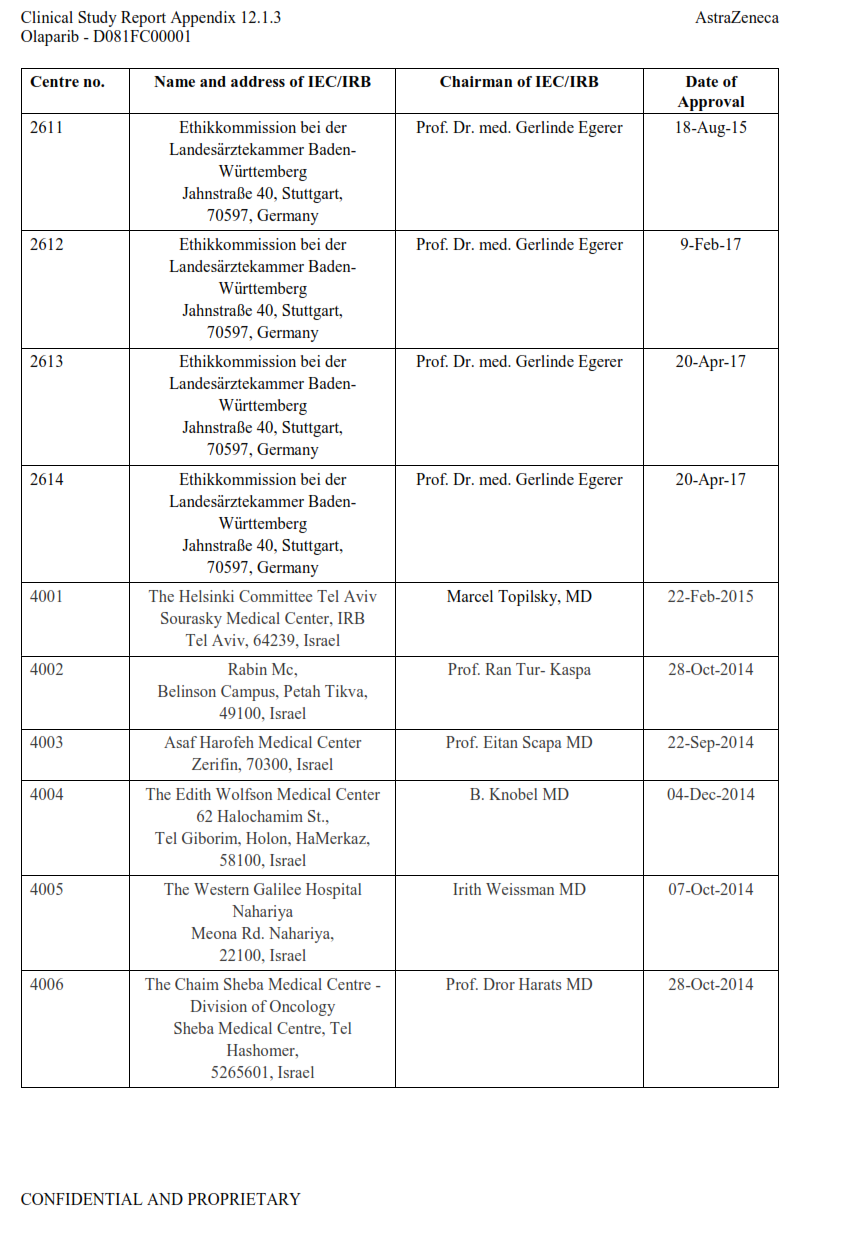

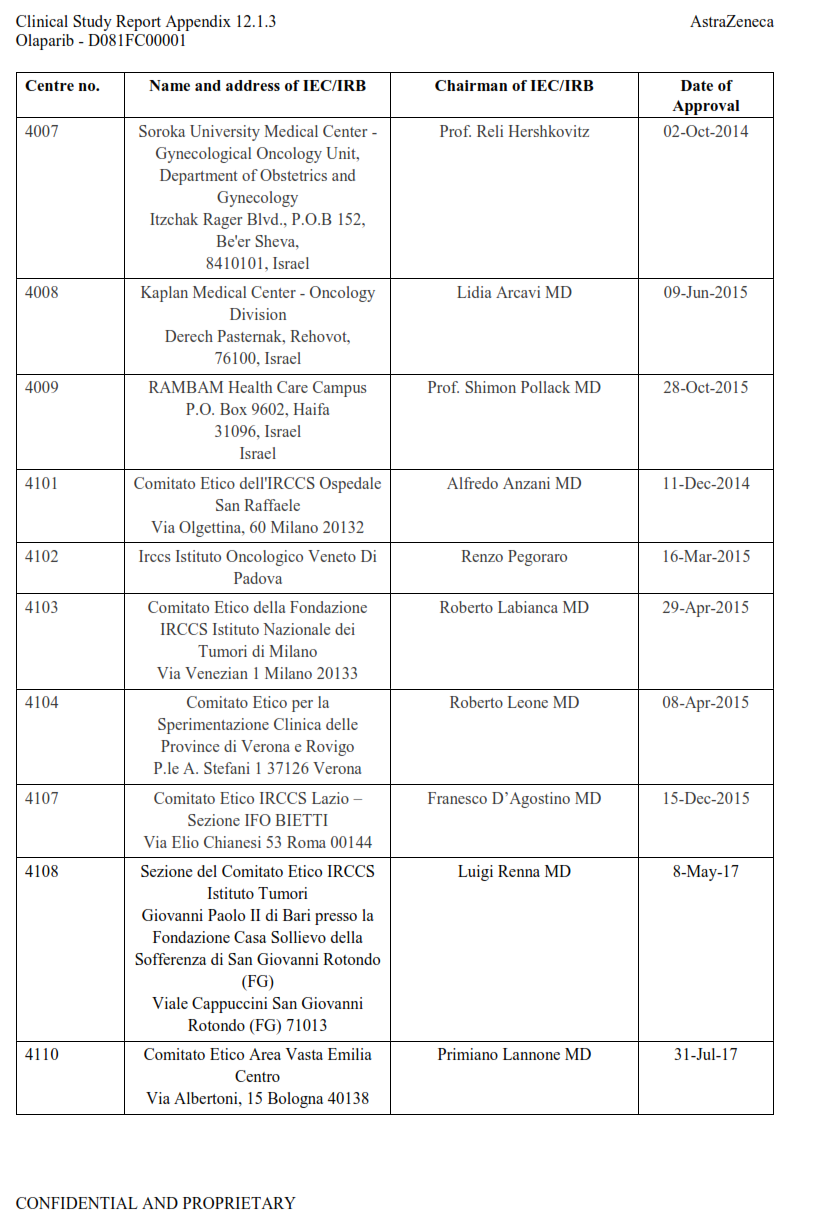

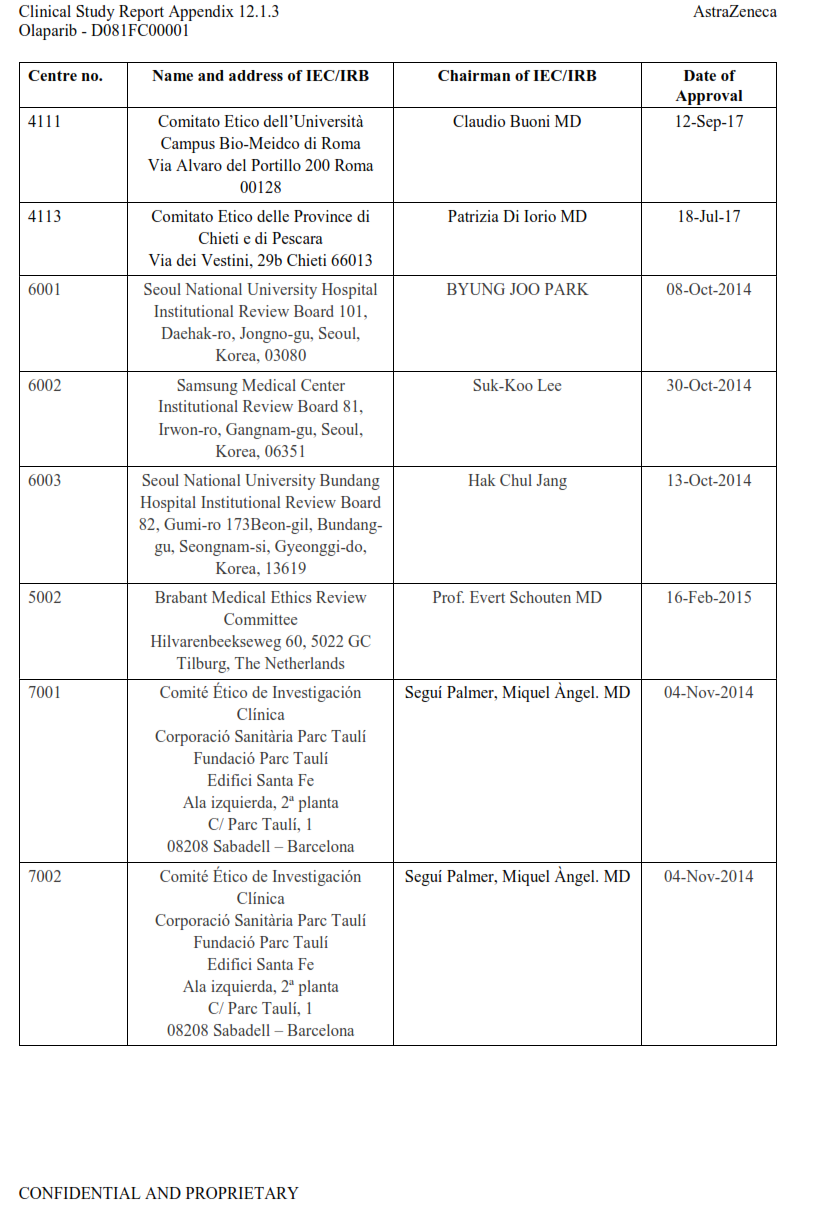

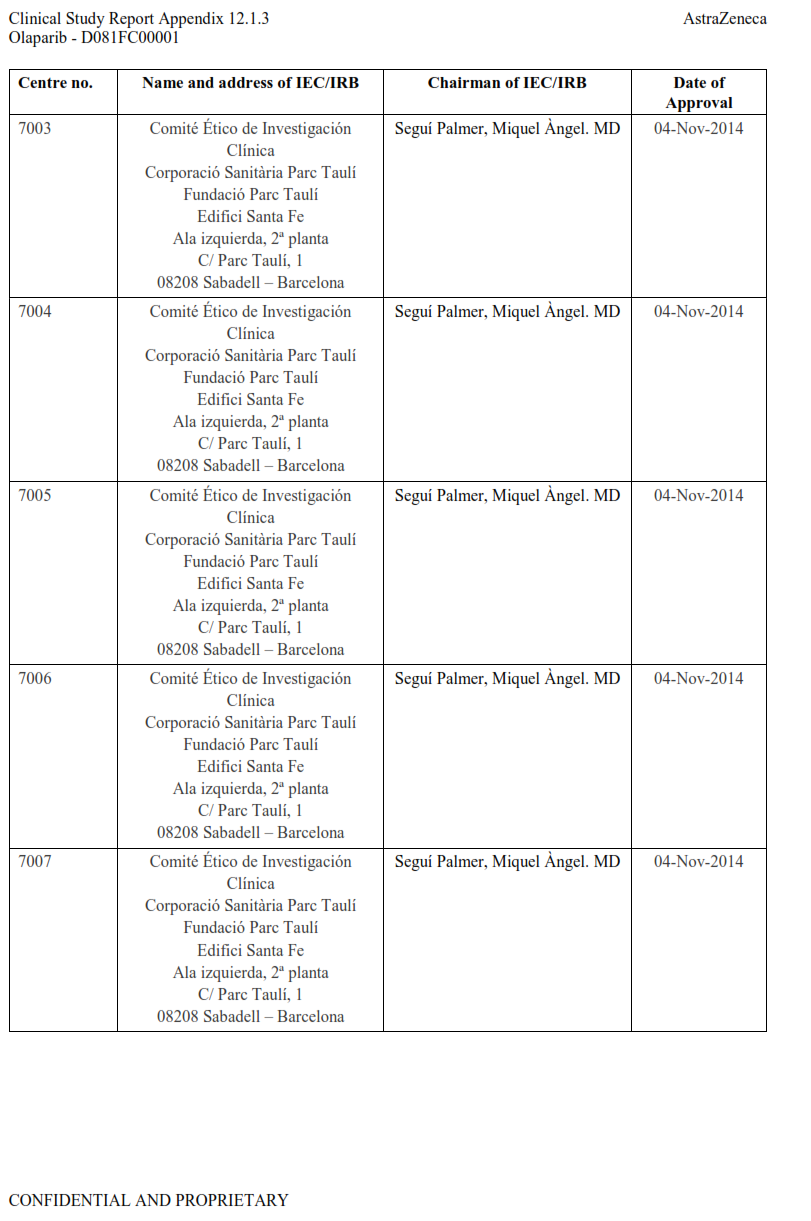

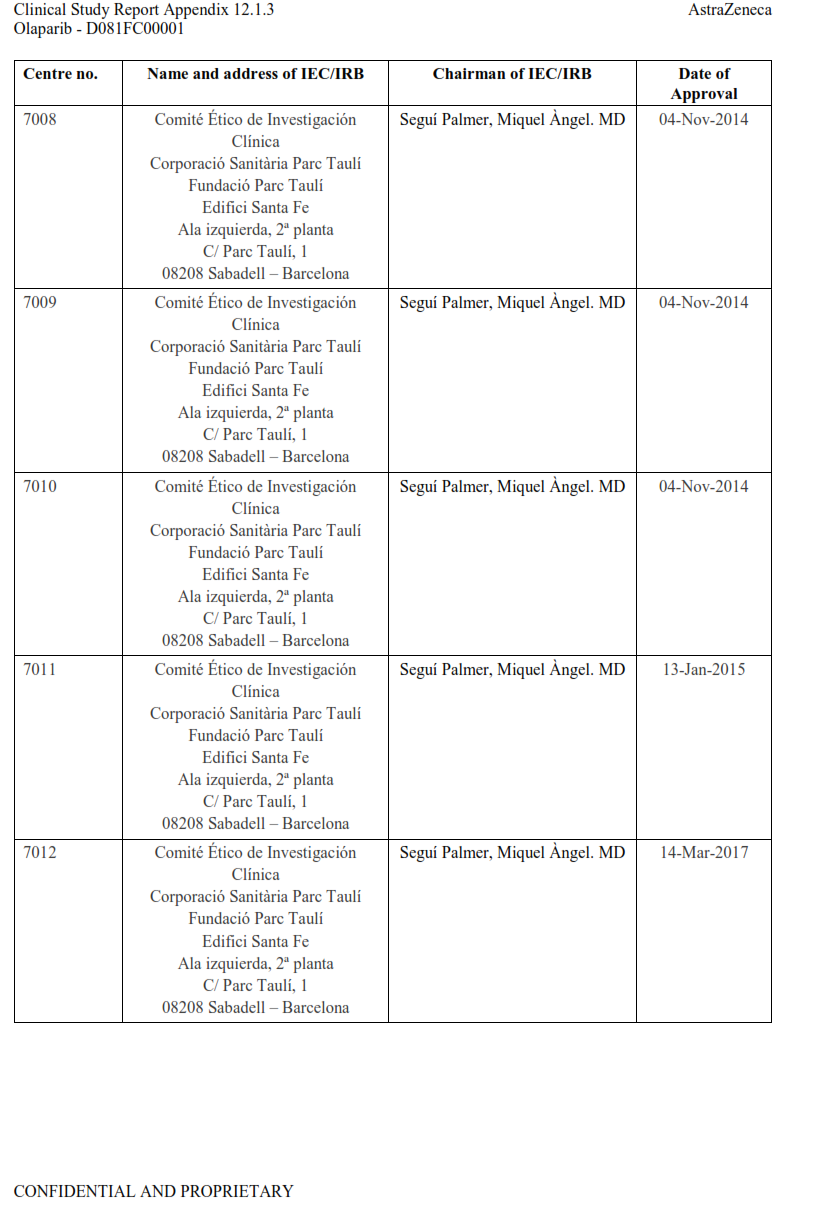

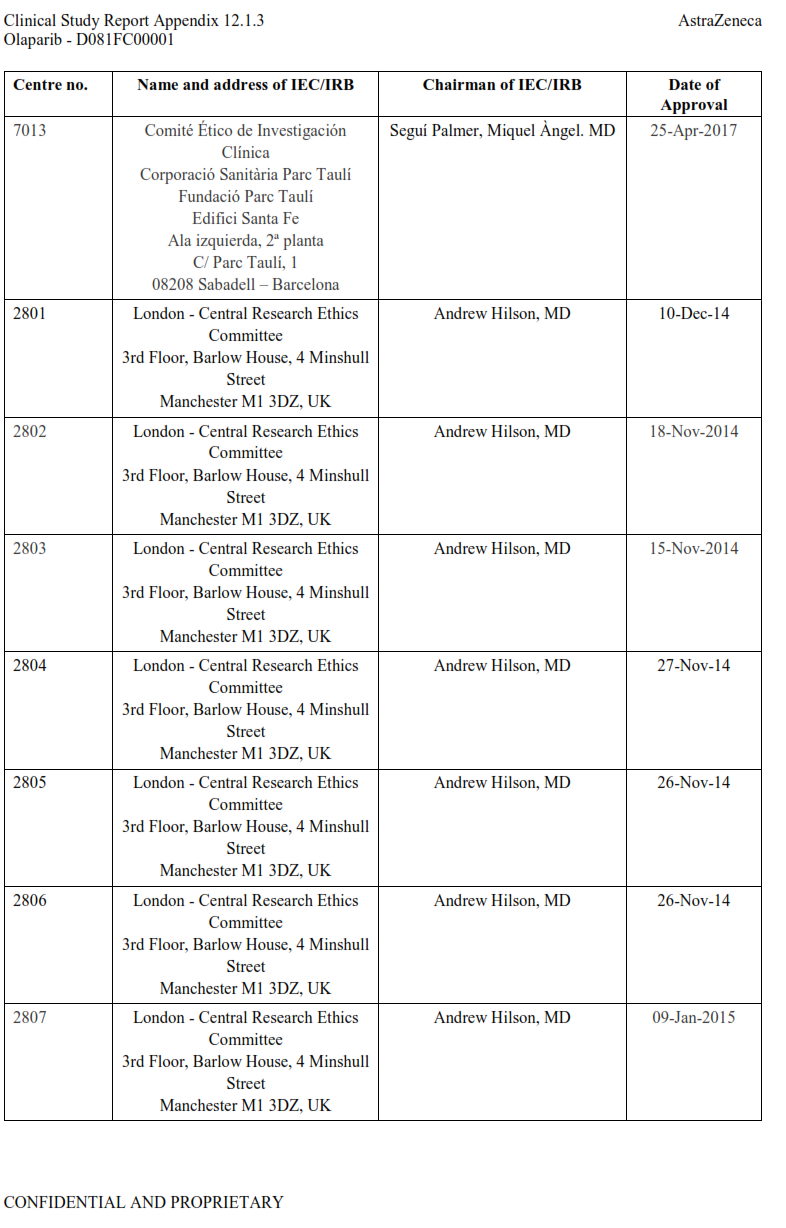

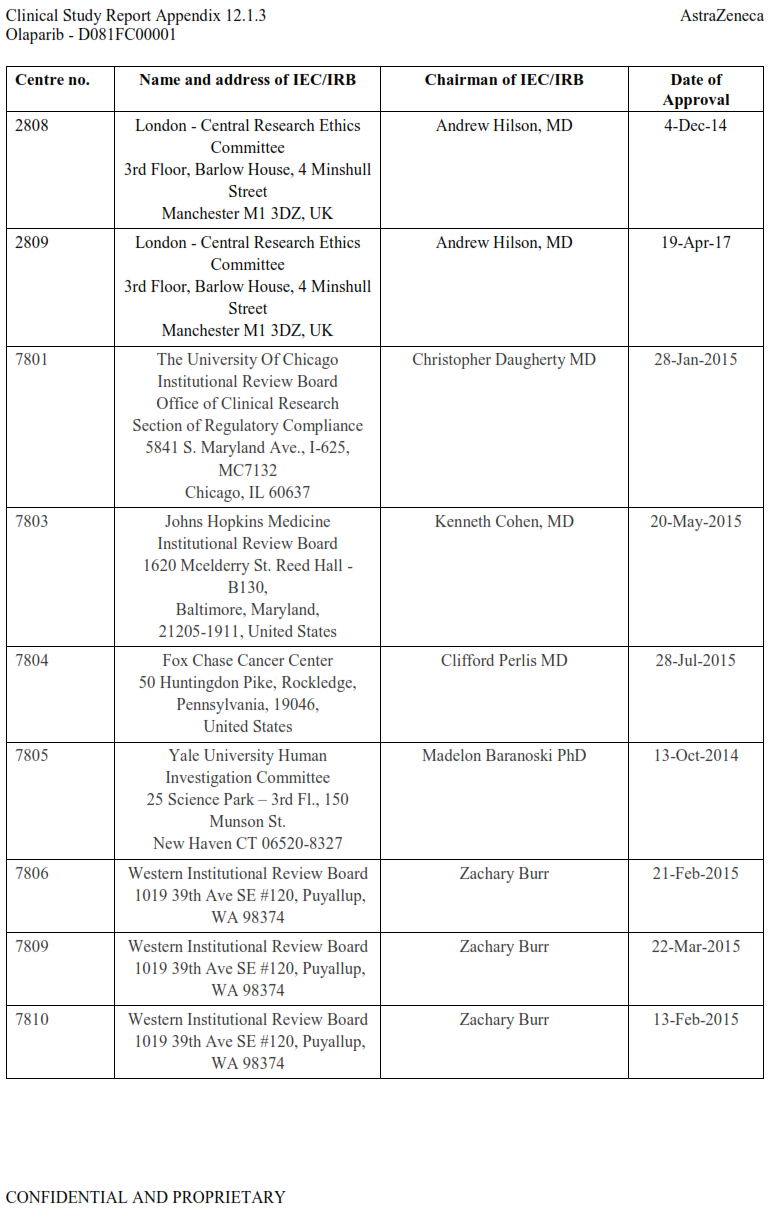

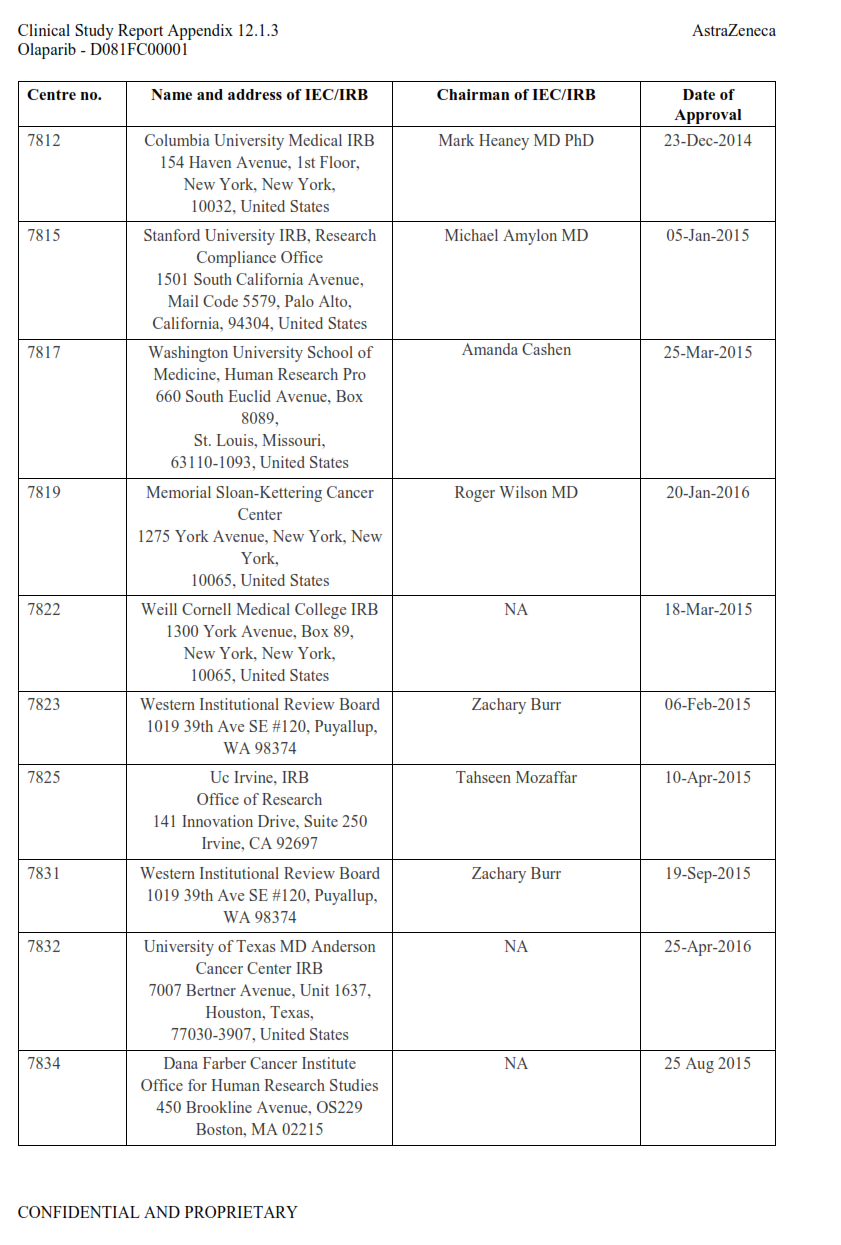

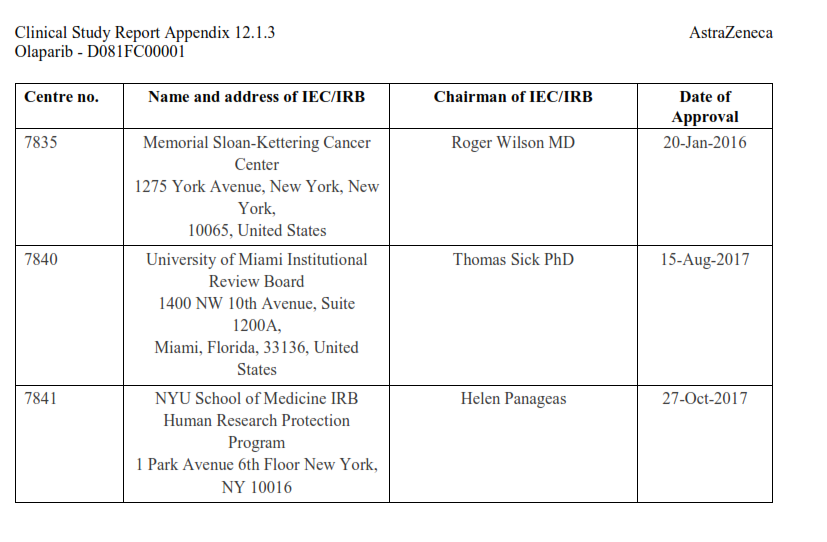

Supplement: Supplementary file 1 — Additional file 1. [file 12885_2022_9661_MOESM1_ESM.docx]
